# Viral tropism and detection of clade 2.3.4.4b H5N8 highly pathogenic avian influenza viruses in feathers of ducks and geese

**DOI:** 10.1038/s41598-021-85109-5

**Published:** 2021-03-15

**Authors:** Nicolas Gaide, Charlotte Foret-Lucas, Thomas Figueroa, Timothée Vergne, Marie-Noëlle Lucas, Luc Robertet, Marie Souvestre, Guillaume Croville, Guillaume Le Loc’h, Maxence Delverdier, Jean-Luc Guérin

**Affiliations:** IHAP, Université de Toulouse, ENVT, INRAE, 23 Chemin des Capelles, 31076 Toulouse Cedex 3, France

**Keywords:** Infectious diseases, Infection, Infectious-disease diagnostics, Influenza virus

## Abstract

Highly Pathogenic Avian Influenza viruses (HPAIVs) display a tissue pantropism, which implies a possible spread in feathers. HPAIV detection from feathers had been evaluated for H5N1 or H7N1 HPAIVs. It was suggested that viral RNA loads could be equivalent or higher in samples of immature feather compared to tracheal (TS) or cloacal swabs (CS). We investigated the suitability of feathers for the detection of clade 2.3.4.4b H5N8 HPAIV in ducks and geese field samples. In the six H5N8 positive flocks that were included in this study, TS, CS and immature wing feathers were taken from at least 10 birds. Molecular loads were then estimated using real-time quantitative reverse transcription polymerase chain reaction (RT-qPCR) targetting H5 and M genes. In all flocks, viral loads were at least equivalent between feather and swab samples and in most cases up to 10^3^ higher in feathers. Bayesian modelling confirmed that, in infected poultry, RT-qPCR was much more likely to be positive when applied on a feather sample only (estimated sensitivity between 0.89 and 0.96 depending on the positivity threshold) than on a combination of a tracheal and a cloacal swab (estimated sensitivity between 0.45 and 0.68 depending on the positivity threshold). Viral tropism and lesions in feathers were evaluated by histopathology and immunohistochemistry. Epithelial necrosis of immature feathers and follicles was observed concurrently with positive viral antigen detection and leukocytic infiltration of pulp. Accurate detection of clade 2.3.4.4b HPAIVs in feather samples were finally confirmed with experimental H5N8 infection on 10-week-old mule ducks, as viral loads at 3, 5 and 7 days post-infection were higher in feathers than in tracheal or cloacal swabs. However, feather samples were associated with lower viral loads than tracheal swabs at day 1, suggesting better detectability of the virus in feathers in the later course of infection. These results, based on both field cases and experimental infections, suggest that feather samples should be included in the toolbox of samples for detection of clade 2.3.4.4b HPAI viruses, at least in ducks and geese.

## Introduction

Avian influenza is a highly contagious infectious disease due to an Influenza A virus belonging to *Orthomyxoviridae* family^1^. Wild birds are considered as the natural reservoir that contributes to the spread of the disease, generating occasionally panzootic with massive death of wild and domestic birds and significant economic losses^2,3^.

During winter 2016–2017, a highly pathogenic avian influenza virus (HPAIV) H5N8, belonging to A/goose/Guangdong/1/1996 clade 2.3.4.4b lineage, caused epidemic outbreaks in Europe^4^. France was the most affected country with more than 6 million culled birds and 484 outbreaks. Free-range foie gras ducks were particularly affected, representing more than 80% of outbreaks^5^. Epidemiological studies pointed out that poor biosecurity practices, interactions with wild birds and transportation of ducks could have contributed to the spread of the virus within this livestock sector^5–9^.

Classical recommendation for the surveillance of influenza involves the collection of tracheal or oropharyngeal and cloacal swabs on live and dead birds. Samples can be processed individually or pooled to detect Influenza A specific nucleic acid signature, by real-time quantitative reverse transcription polymerase chain reaction (RT-qPCR), followed by pathotyping for cleavage site identification^10,11^.

Highly pathogenic avian influenza viruses (HPAIVs) differ from low pathogenic AIVs (LPAIVs) by the mutational acquisition of multibasic haemagglutinin cleavage site, that shifts mucosal epitheliotropism to tissue pantropism, leading to systemic diseases with high mortality in gallinaceous species^3,12^. Since HPAIVs display tissue pantropism, it implies that they could spread in feathers. Accurate detection of HPAIVs from feather samples has been demonstrated for HP Asian H5N1 and H7N1 viruses. These few studies suggest that viral loads detected in immature feathers are equivalent or even higher than those detected on either tracheal or cloacal swabs^13–18^. These results were also confirmed on carcasses of dead birds and detached feathers, suggesting better preservation of viral particles in feathers than in viscera^15,19^. However, almost all these studies were carried out in the context of experimental infections. Combining different approaches, we investigated the suitability of feather samples from both field and experimental infections to detect clade 2.3.4.4b H5N8 HPAIV in ducks.

## Methods

### Field cases sampling

Six flocks were included in the study based on sudden onset of nervous signs and mortality. These flocks have been tested positive for M and H5 AI genes by RT-qPCR^10^. Finally, all these flocks were confirmed positive for 2.3.4.4 H5N8 HPAIV between January and March 2017 by the French National Laboratory for Avian Influenza and Newcastle disease, using the official procedures^10^. They included 4 mule duck flocks (5 to 13 weeks of age), 1 Pekin duck flock (8 weeks of age) and 1 flock of geese (8 weeks of age). In each flock, at least 10 non-clinically affected birds were randomly taken and sampled using tracheal (TS) and cloacal swabs (CS), as well as immature wing feathers (remiges). Mature wing feathers were also collected on geese flock. Skin samples with feathers were harvested following necropsy of clinically-affected ducks (n = 11) and geese (n = 4) and fixed in 10% buffered formalin to investigate viral tropism and feather lesions. On-farm investigations and collection of samples were performed in strict compliance with regulation and biosecurity procedures, with the authorization and supervision of official veterinary services and before implementation of sanitary culling and destruction of carcasses.

### Animal experiment

For the experiment, twenty 10-week-old mule ducks, clinically healthy and AIV negative, were included in the study, in a BSL3 laboratory with negative pressure isolators, absolute air filtration and more than 2 m^2^ surface per isolator. Animals were randomly split into four groups to represent the different modalities of transmission. In the first isolator, five ducks were inoculated at day 0 with 10^6^ EID_50_ H5N8 (A/duck/France/171201g/2017(H5N8)) through choanal instillation (*Infected group*) with additional 5 non-infected ducks (*contact group*). Five non-infected ducks were put in an isolator receiving air from the infected group isolator (*aerosol group*). Five additional non-infected ducks were housed in another isolator into a fully closed system (*control group*). During the seven following days, ducks were fed and had access to water ad libitum. For each animal, tracheal swabs (TS), cloacal swabs (CS), feather pulp (FP) and blood were sampled at days 0 to check negativity (all samples) and then from day 1 to day 7.

### Viral isolation and amplification

To obtain a viral stock for experimental infections, viral isolation and amplification were conducted on feather pulp of an infected mule duck of flock 2 with the highest Cq value on RT-qPCR. For viral isolation, feather pulp was placed in 500 µL of 1X PBS and vortexed for 30 s. Then 150 µL of supernatant at the dilution 1:100 in PBS with penicillin and streptomycin 4X were inoculated to 10-day-old specific-pathogen-free embryonated chicken eggs (INRAE PFIE, Nouzilly, France). Eggs were kept at 37 °C in a humidity chamber during 48 h and then placed at 4 °C overnight. Allantoic liquids were harvested and titrated by hemagglutination assay with fresh and washed chicken red blood cells.

For viral amplification, a similar passage on embryonated eggs was done on allantoic liquid with the highest hemagglutination assay titer. Embryonated chicken eggs were inoculated with 100 µL of a 1:100 dilution in PBS with penicillin and streptomycin 2X. The viral stock was then aliquoted and stored at − 80 °C. In parallel, the titer of this viral stock was also determined by Tissue Culture Infectious Dose (TCID50) method on MDCK cells and calculated by the Spearman & Kärber algorithm^20^. All these steps were processed in a BSL3 lab in strict compliance with biosafety procedures. Sequences of amplified A/duck/France/171201g/2017(H5N8) virus, first isolated from a mule duck feather, were deposited on GenBank database under MK208604-MK208610 accession numbers.

### Histopathology and immunohistochemistry

Formalin-fixed cutaneous tissues were routinely processed in paraffin blocks, sectioned at 4 μm and stained with hematoxylin and eosin (HE) for microscopic examination. A diagram representing histological structures of avian feathered skin is provided in supplementary data (Suppl. Fig. S1). The number of follicular sections examined per skin section was 8 in average for ducks (n = 11 birds) and 4 for geese (n = 4 birds). Immunostainings were performed on paraffin-embedded skin sections with a monoclonal mouse antibody against Influenza A virus nucleoprotein (NP) (Argene, Sherley, USA, 11–030). Immunohistochemical reaction included an antigen retrieval with pronase 0,05% 10 min at 37 °C, a peroxidase blocking step 5 min at room temperature (Dako, Glostrup, Denmark, S2023) and saturation of non-specific binding sites with normal goat serum (Dako, Glostrup, Denmark, X0907) 25 min at room temperature, before overnight incubation at 4 °C with anti-NP antibody at the dilution 1:50. The immunohistochemical staining was revealed with a biotinylated polyclonal goat anti-mouse immunoglobulins conjugated with horseradish peroxidase (HRP) antibody (Dako, Glostrup, Denmark, LSAB2 system-HRP, K0675) and the aminoethylcarbazole chromogen of the HRP (Dako, Glostrup, Denmark, AEC + Substrate-Chromogen, K3469). Negative controls included sections incubated either without specific primary antibody or with another monoclonal antibody of the same isotype (IgG2). Histopathological analyses were carried out by three veterinary pathologists certified by the European College of Veterinary Pathologists (ECVP).

### RNA extraction and RT-qPCR

All samples were processed in BSL3 laboratory until lysis in strict compliance with biosafety procedures. Swabs were placed in 500 µL of 1X PBS and vortexed for 30 s. Feather pulp was stored in 500 µL of 1X PBS and then vortexed for 30 s before RNA extraction. For experimental infected group, additional 30–60 mg feather pulp were concurrently incubated in 200 µL PBS 1X containing 0,8 µg/µL of proteinase K (Thermo fisher #EO0492) for 20 min at 37 °C to compare RNA extraction yields with and without proteinase K. For all sample types, RNA was extracted from 140 µL of supernatant with QIAamp Viral RNA Mini Kit (Qiagen #52906) and then kept at − 80 °C.

RT-qPCR was performed on 2 µL RNA using iTaq Universal SYBR Green Supermix (Bio-Rad #172–5125). M and H5 genes were targeted with M52C / M253R primers for M gene^21^, and H5_HP_EA_F2 (5′-TCCTTGCAACAGGACTAAG-3′) / H5_HP_EA_R (5′-GTCTACCATTCCYTGCCA-3′)^22^. Absolute quantification was performed with a plasmid range from 10^2^ to 10^7^ copies/reaction (2 µL). RT-qPCR reaction and results interpretation were performed on a LightCycler 96 instrument (Roche).

### Droplet digital PCR

For cDNA synthesis, 5 µL of RNA extracted from swabs and feather pulp (from field cases flock 2) were processed according to the instructions of the RevertAid First Strand cDNA Synthesis Kit (Thermo Scientific #15255146). As negative controls, 5 μL of Milli-Q water was processed in parallel. A 2 μL aliquot of the cDNA from the reverse transcription step of samples and controls were used for PCR amplification with the QX200 ddPCR EvaGreen Supermix (Bio-Rad 1864033) and M and H5 primers previously described. ddPCR were processed according to the instruction for QX200 AutoDG Droplet Digital PCR System (Bio-Rad) established by the Transcriptomic platform from Genotoul (Get-Santé INSERM I2MC Toulouse). Analyses were performed with the dedicated software Quantsoft (Bio-Rad).

### Bayesian data analysis

To estimate the sensitivity and the specificity of detection in the three different sample types (cloacal swab, tracheal swab and feather pulp) at the individual level, we used a latent class modelling approach embedded in a Bayesian framework. This type of analysis has been extensively used to model cross-detection of individuals whose true epidemiological status (infected or not) is assessed using imperfect diagnostic tests of unknown sensitivity and specificity^23^. To do so, we defined five thresholds of test positivity (5, 10, 20, 50 and 100 copies of RNA/μL), and derived the observed frequency of the 2^3^ = 8 different combinations of individual test results for each threshold. The combination of test results was assumed to be distributed according to a multinomial distribution of parameters n = 61 birds and eight probabilities, expressed as a combination of seven parameters to be estimated, i.e. the proportion of infected birds in the sample and the sensitivity and specificity of detection in the three different sample types. The sensitivity and specificity parameters were assumed to be threshold-specific (leading to 30 parameters, 6 for each of the five thresholds of positivity) while the proportion of infected birds was considered the same irrespective of the threshold. The value of the 31 parameters was estimated in a Bayesian framework. We allocated uniform prior distributions for the proportion of infected birds and all sensitivity parameters. All specificity parameters were assigned an informative beta prior distribution to reflect that we were 97.5% and 50% confident that the true value of the specificity parameters were greater than 0.8 and 0.98, respectively.

### Approval for animal experiments

This study was carried out in compliance with European animal welfare regulation. The protocols were approved by the Animal Care and Use Committee “Comité d’éthique en Science et Santé Animales—115”, protocol number 13205-2018012311319709.

## Results

### Integument lesions and virus detection of field cases in H5N8-infected ducks and geese

**Figure 1:**
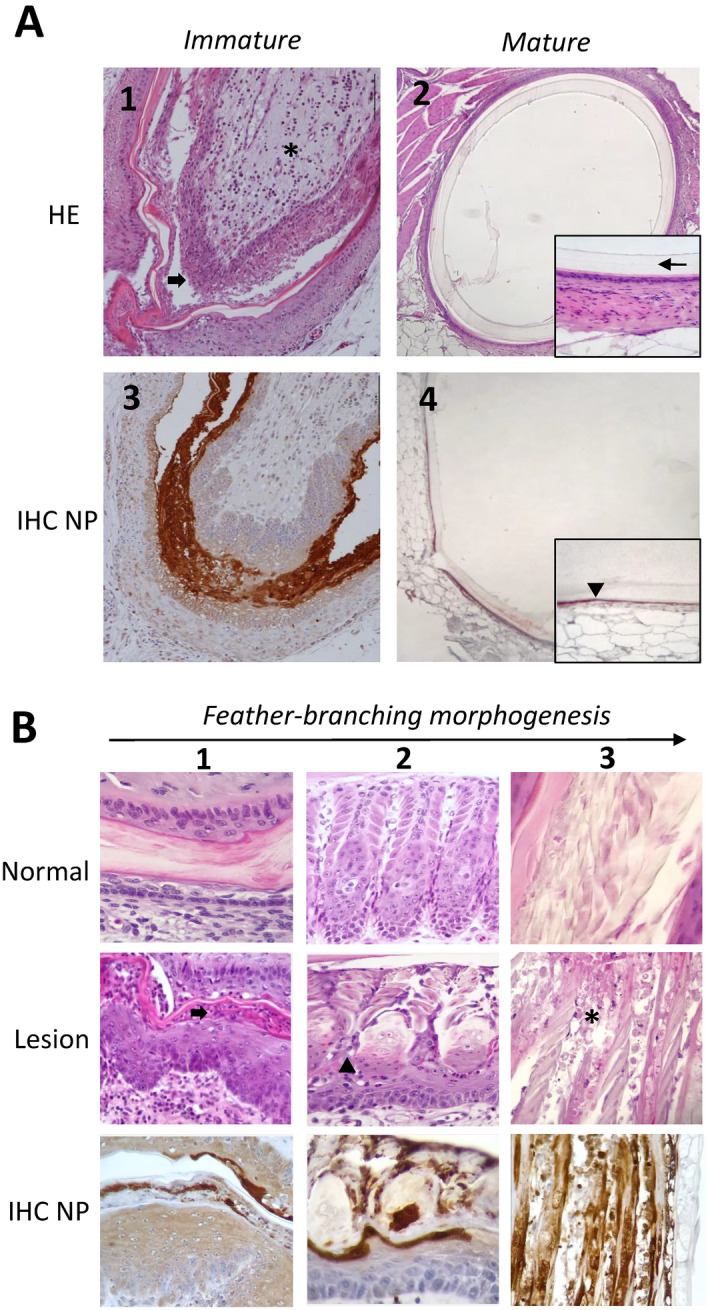
Lesion and viral detection in immature and mature feathers of naturally H5N8-infected ducks and geese. Hematoxylin and Eosin (HE) and anti-nucleoprotein A Immunohistochemistry (IHC). (**A**) Lesion and viral detection in immature and mature feathers, duck infected with H5N8 clade 2.3.4.4b, HE. (**A1**) Immature feather with extensive acute necrosis of feather and follicular epidermis (arrow). The feather pulp is infiltrated by leukocytes, mainly lymphocytes and plasma cells (asterisk). (**A2**) Mature feather with calamus (thin arrow) within normal limits. (**A3**) Abundant viral antigen is present within necrotic debris and outer layers of feather and follicular epidermis. (**A4**) Viral antigen is present in association with follicular epidermis of mature feather, under the calamus (arrowhead). (**B**) Growing and differentiating feather epidermis, duck infected with H5N8 clade 2.3.4.4b. (**B1**) At early stage of differentiation, feather epidermis is stratified. Necrosis is visible at the outer (corneal) layer (arrow) with viral antigen colocalization. (**B2**) At the stage of early differentiation, necrosis and single-cell necrosis/apoptosis can be seen within marginal plates and barb ridges (arrowhead) and with viral antigen colocalization. (**B3**) At the stage of cornication, keratinized barbs and barbules are disrupted and intermixed with necrotic cells and debris (asterisk) with viral antigen colocalization.

Duck skin sections consisted of a mixture of immature and mature feathers, whereas geese presented a majority of immature feathers. Lesions were observed in immature feathers in 91% of ducks and 50% of affected geese (Table 1, Fig. 1A). Mature feathers were within normal limits for all subjects. In ducks, lesions were consistent with focal to extensive necrosis of feather (64%) and follicular (73%) epidermis in association with leukocytic infiltration of feather pulp (91%), including predominantly lymphocytes and plasma cells, and fewer macrophages and heterophils. Necrosis was predominant in the outer (corneal) layer and could be observed from early to late stage of feather epithelium differentiation, including early and late barb ridges and marginal plates (Fig. 1B). Variable amount of leukocytic infiltration including lymphocytes, plasma cells and heterophils were observed in the follicular dermis in 73% of ducks. In geese, lesions were less frequent: half of the subjects presented leukocytic infiltration of pulp and necrosis of feather epidermis (50%) and one subject presented epidermal focal necrosis in follicular epidermis (25%).

**Table 1:** Proportion of naturally H5N8-infected ducks and geese with histopathological lesions and positive viral detection in immature and mature feathers.

	Immature feather				Mature feather
	Feather dermis	Feather epidermis	Follicular epidermis	Follicular dermis	
**Histopathology**^**a**^					
Duck n = 11	10/11 (91%)^b^	7/11 (64%)	8/11 (73%)	8/11 (73%)	0/11 (0%)
Goose n = 4	2/4 (50%)	2/4 (50%)	1/4 (25%)	0/4 (0%)	0/4 (0%)
**Immunohistochemistry**^**c**^					
Duck n = 11	7/11 (64%)	11/11 (100%)	11/11 (100%)	8/11 (73%)	10/11 (91%)^d^
Goose n = 4	0/4 (0%)	4/4 (100%)	3/4 (75%)	0/4 (0%)	NE

*NE* non evaluated.

^a^Focal to extensive lytic necrosis and apoptosis/single cell necrosis in epidermal structures. Mixed leukocytic lympho-plasmacytic rich infiltration in dermal structures.

^b^Total number of positive subjects / Total subjects (percentage of positive subjects).

^c^Anti-nucleoprotein A immunohistochemistry.

^d^Positive antigen detection in follicular epidermis.

In immature feathers, viral antigen was detected in all ducks and geese samples, mainly in feather and follicular epidermis. Viral antigen immunostaining was patchy to diffuse in distribution and severely intense in the outer (corneal) layer epidermis and necrotic areas (Fig. 1A). Interestingly, viral antigen was commonly detected at all levels of feather epithelial differentiation in ducks and geese, including barb ridges, marginal plate, barbs and barbules (Fig. 1B). In mature feathers, 91% of ducks presented immunostaining of follicular epidermis (corneal layer) without associated lesions (Fig. 1A). In ducks, viral antigens were occasionally detected in mesenchymal cell in feather pulp (64%) and follicular dermis (73%).

**Figure 2:**
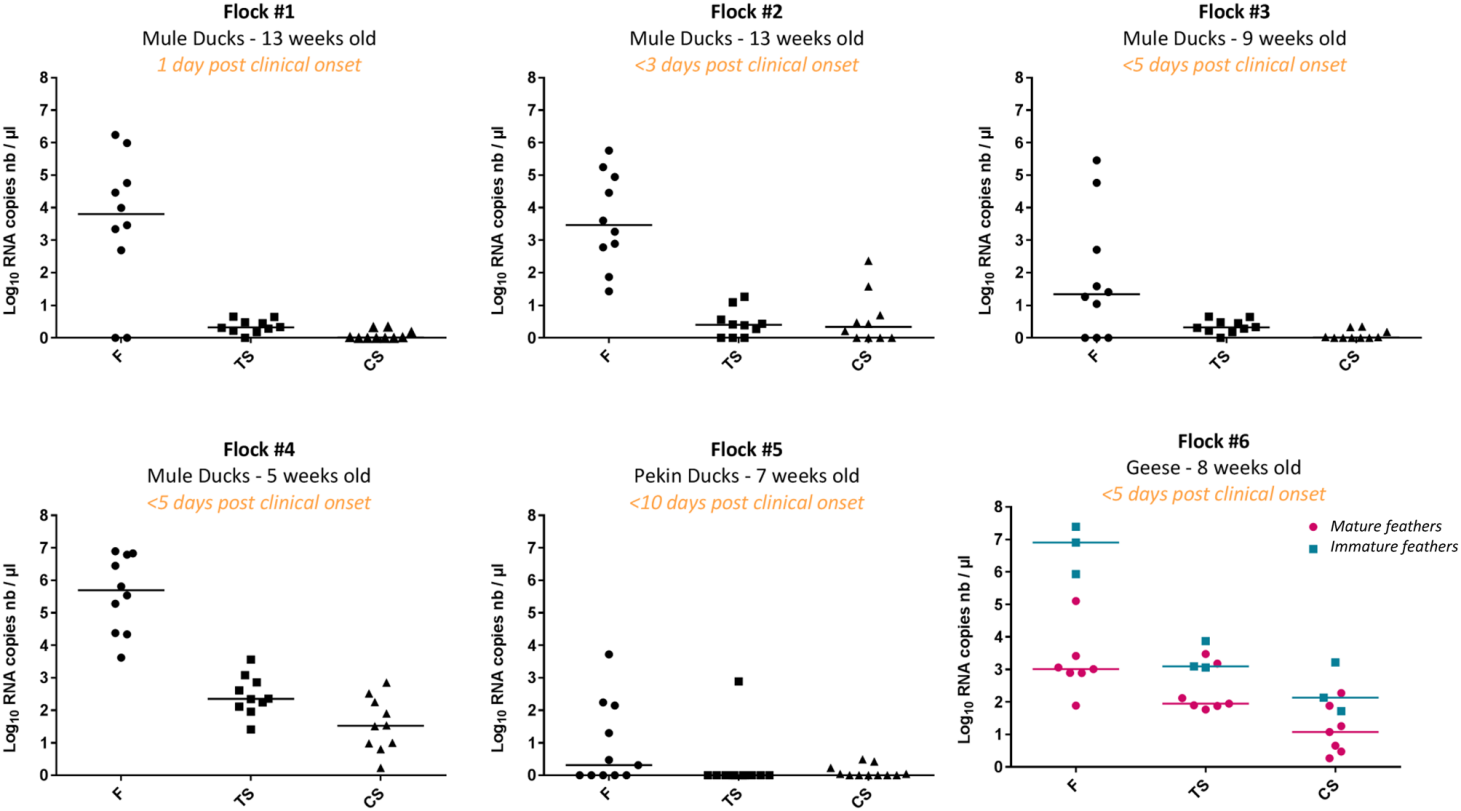
Viral RNA loads in feathers (F), tracheal swabs (TS) and cloacal swabs (CS) in 6 naturally infected flocks with H5N8 HPAIV. Mule ducks (4 flocks, n = 10 birds per flock), pekin ducks (1 flock, n = 11) and geese (1 flock, n = 10). Results are expressed as log_10_ viral RNA copies/µL.

**Figure 3:**
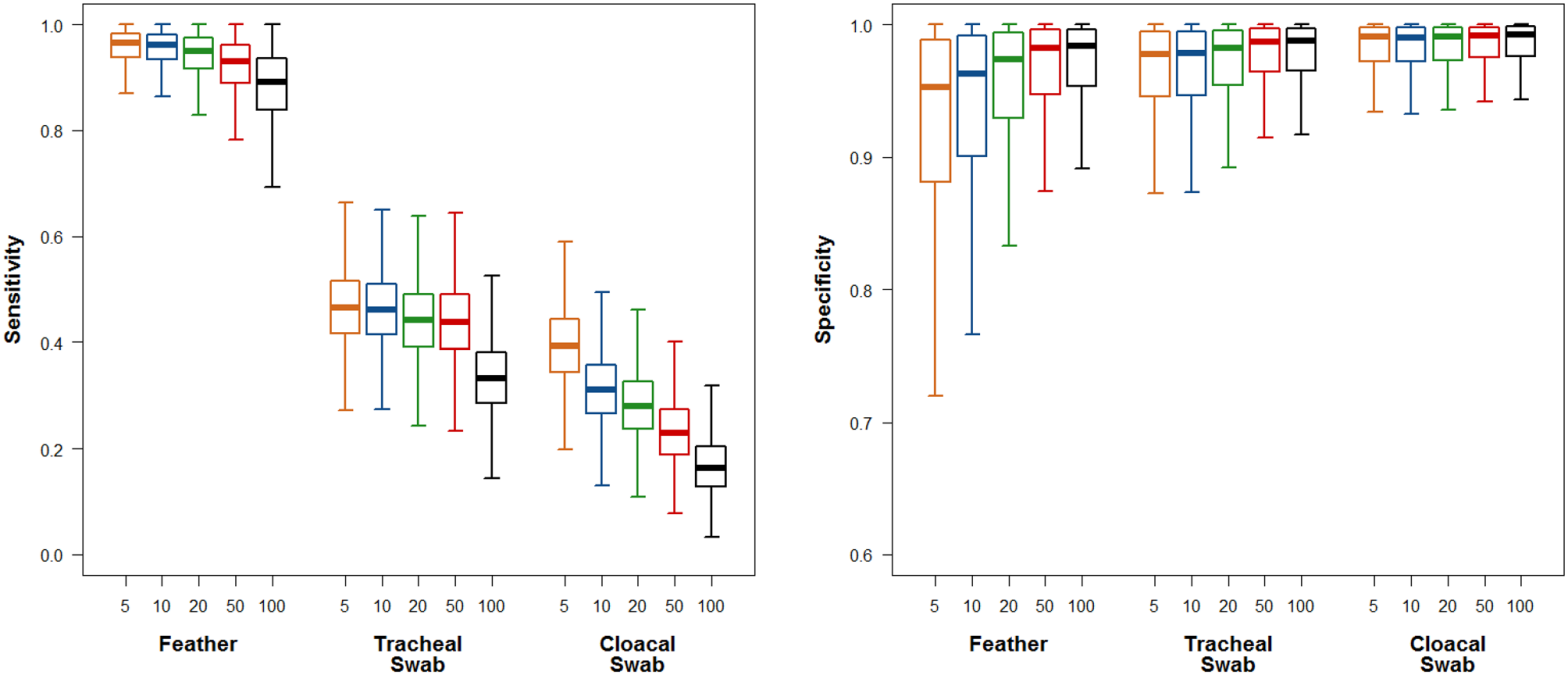
Estimated sensitivity (left panel) and specificity (right panel) of the three different sample types (feather sample, tracheal swab and oropharyngeal swab) for 5 different thresholds of positivity (5, 10, 20, 50 and 100 copies of RNA/µL).

In all six duck flocks included in the study, RNA loads of HPAIV in feather pulp were at least equivalent and in most cases up to 10^3^ higher than those detected in either TS or CS (Fig. 2). Incubation of swabs with proteinase K was assessed on a few samples and did not result in a significant increase in detectability (Suppl. Fig. S2). For a given positivity threshold, feather pulp tested positive much more frequently than TS or CS (Suppl. Table T1). A latent class modelling approach was used to estimate the sensitivity and specificity of RNA detection in each sample type (feather pulp, CS and TS). Irrespective of the threshold of positivity (5, 10, 20, 50 and 100 copies of RNA/μL), the sensitivity of RNA detection by RT-qPCR in feather pulp of infected birds was much higher than in TS or CS, while the specificity did not substantially drop (Fig. 3). As an example, for a positivity threshold of 10 copies of RNA/mL, the sensitivity of the detection in feather pulp was estimated at 0.96 (95% credible interval (95% CI) 0.86–1.00), while the sensitivity of detection in tracheal swabs and cloacal swabs were estimated at 0.46 (95% CI 0.32–0.60) and 0.31 (95% CI 0.19–0.45), respectively.

As shown in Table 2, testing one sample of feather pulp was even shown to be more sensitive (Se = 0.96 (95% CI 0.86–1.00)) than the classical sampling recommendation, involving the combination of one tracheal swab and one cloacal swab (Se = 0.63 (95% CI 0.51–0.75) for a positivity threshold of 10 RNA copies/mL). To further confirm the quantitation in tissues, a Droplet Digital PCR assay, using the same primer set, was implemented on a subset of samples. This assay showed a substantial correlation with RT-qPCR data and confirmed significant viral loads in feathers (Suppl. Fig. S3).

**Table 2:** Comparison of the detectability of the infection for each threshold of positivity between a sampling strategy involving one feather only and a sampling strategy involving a tracheal swab and an oropharyngeal swab (in which case an animal tested positive is defined by a positive result for at least one of the two swabs).

Parameter	Threshold of positivity (nb copies of RNA/μL)	1 feather sample	1 tracheal and 1 oropharyngeal swabs
Sensitivity	5	0.96 (0.87–1.00)	0.68 (0.56–0.80)
	10	0.96 (0.86–1.00)	0.63 (0.51–0.75)
	20	0.95 (0.83–1.00)	0.60 (0.47–0.72)
	50	0.93 (0.79–0.99)	0.57 (0.44–0.70)
	100	0.89 (0.73–0.99)	0.45 (0.31–0.59)
Specificity	5	0.95 (0.68–1.00)	0.96 (0.82–1.00)
	10	0.96 (0.71–1.00)	0.96 (0.81–1.00)
	20	0.97 (0.78–1.00)	0.96 (0.83–1.00)
	50	0.98 (0.82–1.00)	0.97 (0.85–1.00)
	100	0.98 (0.83–1.00)	0.97 (0.85–1.00)

The brackets represent the 95% credible interval of the posterior distributions.

### Dynamics of HPAIV detection in feathers and swabs in experimentally infected ducks

**Figure 4:**
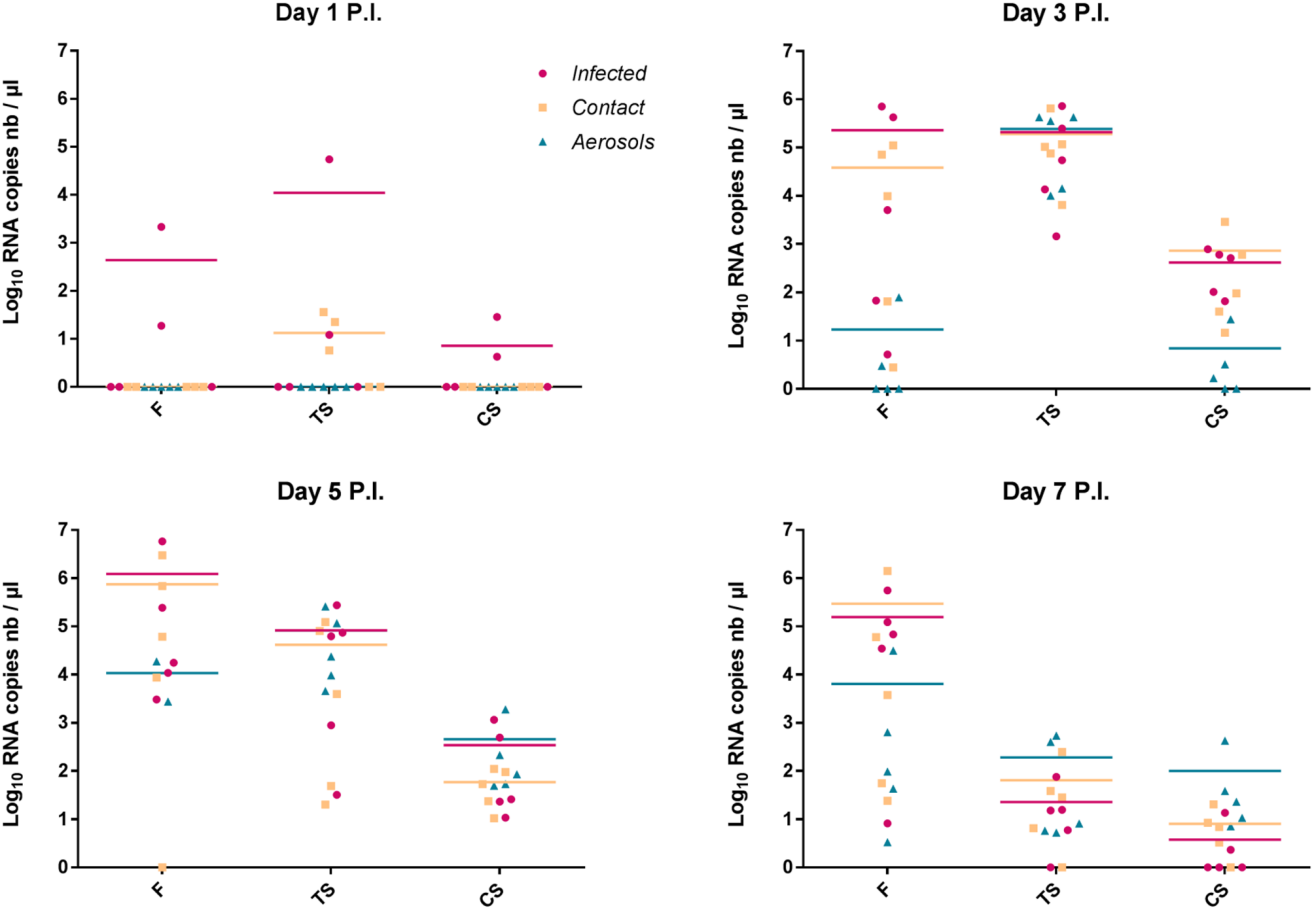
Viral RNA loads in experimentally H5N8-infected 10-week-old mule ducks.Virus loads in feathers (F), tracheal swabs (TS) or cloacal swabs (CS) were determined from day 1 to day 7 post-infection for infected (pink), contact (yellow) and aerosol (blue) groups. Absolute quantification was performed by RT-qPCR targeting the viral M gene from extracted RNAs in parallel with an M gene plasmid range. Each dot represents an individual value and horizontal bars correspond to means. limit of detection for each experiment.

Experimental infection resulted in totally asymptomatic infections in all birds, despite viral RNA detection in swabs and feathers starting from day 1 post-infection (Fig. 4) and RT-qPCR Ct values as low as 16 in some birds at peak of infection (data not shown). Control birds remained virus negative throughout the study. Three direct contact ducks became positive in tracheal swabs as early as day 1 post-infection and by day 3 post-infection, they were all positive in all sample types. Regarding birds connected through aerosols, some became positive at day 3 post-infection, with two positive feather samples, five positive tracheal swabs and three positive cloacal swabs, and they were all positive in all sample types by day 5 post-infection. Viral loads were always substantially higher in tracheal than cloacal swabs. For feather samples, viral loads were equivalent or substantially higher than tracheal and cloacal swabs at day 3, 5 and 7 post-infection, but not at day 1 post-infection when compared with tracheal swabs of infected and contact groups (Fig. 4). The peak of viral excretion occurred between day 3 and 5 post-infection and the relative viral loads in feathers increased, becoming obviously higher than tracheal and even more, cloacal swabs from day 5 post-infection until the end of the course of infection (Fig. 4).

## Discussion

The present study investigated the suitability of HPAIV H5N8 clade 2.3.4.4b viral detection in feathers with histopathological correlates in ducks and geese. Previous studies focused on the pathology and detectability of H5N1 and H7N1 HPAIVs in the integument after experimental infections of ducks, chicken and geese^13–19^. To the best of our knowledge, the present data provide new insights about HPAIV detection in feather for H5N8 clade 2.3.4.4b through both naturally occurring field cases and experimental challenges.

Our data support previous findings that HPAIVs can replicate in feathers and induce feather folliculitis. Ducks presented epithelial necrotizing folliculitis in immature (growing) feathers, with concurrent viral antigenic detection. This lesional pattern supports a strong tropism for feather and follicular epidermis, corneal outer layers in particular, confirming previous descriptions^24–26^. In 91% of ducks, a marked leukocytic, lymphoplasmacytic rich, infiltration was observed in the feather pulp, which contrasts with previous experimental infections of ducks with Indonesian strain of H5N1, where no inflammatory lesions were found in association with epithelial necrosis^24^. Viral detection in leukocytes was uncommon, more frequent in mesenchymal stromal cells of dermal structures as previously reported^25^. In geese, no major lesion pattern dominated and half of the subjects had feathers within normal limits.

Interestingly, cell death with concomitant viral antigens could be detected at different stages of feather epithelial differentiation, from the early stage up to cornifying barbs, barbules of ducks and geese. Because viral replication seems to occur into feather structures intended to be external, these data suggest that growth and differentiation of feathers epidermis could be a source of viral production for pantropic H5N8 Clade 2.3.4.4b and could potentially carry and disseminate viral particles. The role of feathers in environmental dissemination of avian influenza viruses has been suggested, including in wild waterfowl^19,27^. Viral infectivity and RNA detectability in feathers detached from bodies has been experimentally investigated in ducks and revealed that HPAIV (H5N1) can longer persist compared to drinking water and feces, for 160 days at 4 °C and 15 days at 20 °C^19^. Additionally, a study conducted on wild waterbirds has suggested that preen oil increases AIVs survivability on feathers and can act as protection for viral particles^27^. Altogether, these data indicate that feathers could be a source of environmental contamination. It would be interesting to compare feather infectivity potential between species, since we can presumably predict higher infectivity for species showing an epitheliotropic viral expression in feather compared to chicken where viral expression is endotheliotropic and restricted to the dermal pulp, confined in the calamus. Further studies are needed to confirm this significance of feathers in the epidemiology of avian influenza.

Viral loads and tropism may vary greatly during the course of infection in a same bird, which could impact greatly the efficiency of different samples^15^. In our field investigations, flocks were sampled at day 1 to 7 after the onset of clinical signs and/or mortality, as notified by the farmer or the attending veterinarian. In all batches, viral loads in feathers were higher than in swabs, regardless of this estimated stage of infection. In controlled conditions, as during our experimental infection, viral loads in feathers increased during infection, suggesting that feathers may not be the most relevant samples at the very early stage of the infection (day 1 post-infection).

Classical recommendation for the surveillance of influenza involves the collection of tracheal or oropharyngeal and cloacal swabs on live and dead birds^10,11^. According to our results, the sensitivity of combining one tracheal and one cloacal swab to test for the presence of viral RNA in an infected duck would be 0.63 (0.51–0.75) for a positivity threshold of 10 copies/mL. It is striking that only one feather pulp sample outperform the classical sample recommendation, as its sensitivity was estimated at 0.96 (0.86–1.00). Moreover, feathers are easy to collect, transport and treat for molecular analyses. The much higher sensitivity of detection in feather pulp suggests that feather samples could be diluted without decreasing the overall sensitivity at the flock level, and therefore that they could be pooled further, which would reduce the costs of analysis at the flock scale and improve the overall economic efficiency of surveillance. Combined with swabs in a sampling strategy, feathers would also provide supplementary information about invasiveness of the virus. In a previous experimental study conducted on european quails, H5N1 and H7N1 HPAIVs inoculation resulted in viral RNA detection in feathers from day 1 up to day 6 and day 12, respectively. In contrast, H7N2 LPAIV detection remained negative during all the experiment from 1 to 15 days post-infection^28^. Assuming that AIV spread in feather is the result of viremia and systemic infection, feather folliculitis in combination with positive viral detection could presumably reflect the tissue pantropism of a virus strain and suggest infection by a HPAIV. Further investigations are needed to investigate feather infection as a signature of HPAIVs, including variations between strains.

Clinical expression of HPAI in ducks is poor, as evidenced on the field and in experimental infections^29^. Our experiment confirmed that ducks may excrete huge loads of virus for days, contaminate very efficiently the rest of the flock without expressing clinical signs. This poor clinical expression in ducks stresses, even more, the pivotal importance of an optimized surveillance strategy, based on intensive testing, at least before farm-to-farm transportation of ducks.

These data, based on a selection of both field and experimental cases of H5N8-infected ducks and geese, suggest that feather samples should be included in the toolbox of samples for detection of clade 2.3.4.4b HPAI viruses, at least in ducks. Further investigations are needed in experimental settings and on a wide range of HPAIVs to ascertain the relevance of their insertion in the diagnostic protocols of HPAI surveillance.

## Supplementary Information

Supplementary Information.

## Data Availability

The datasets generated during and/or analysed during the current study are available from the corresponding author on reasonable request.
