# Supplementary material for: Viral tropism and detection of clade 2.3.4.4b H5N8 highly pathogenic avian influenza viruses in feathers of ducks and geese
Source: Sci Rep. 2021 Mar 15;11:5928. doi: 10.1038/s41598-021-85109-5 (PMC7960704; doi:10.1038/s41598-021-85109-5)

Supplementary material

Title: Viral tropism and detection of clade 2.3.4.4b H5N8 highly pathogenic avian influenza viruses in feathers of ducks and geese

Nicolas Gaide[1]*, Charlotte Foret-Lucas[1]*, Thomas Figueroa[1], Timothée Vergne[1], Marie-Noëlle Lucas[1], Luc Robertet[1], Marie Souvestre[1], Guillaume Croville[1], Guillaume Le Loc'h[1], Maxence Delverdier[1] & Jean-Luc Guérin[**]

[1]IHAP, Université de Toulouse, ENVT, INRA, Toulouse, France

*NG and CFL contributed equally and should be both considered as 1st authors

**Author for correspondence: Jean-Luc Guérin, ENVT, 23 Chemin des Capelles 319076 Toulouse Cedex 3 FRANCE. E-mail: jean-luc.guerin@envt.fr

Suppl Table 1. Distribution of the eight cross-classified results of the three sample types from field cases (feather pulp, tracheal swab and cloacal swab).

Positivity in feather pulp	***Positivity in tracheal swab***	***Positivity in cloacal swab***	Number of ducks for the different positivity thresholds				
			5 copies of RNA/µL	10	20	50	100
0	***0***	***0***	11	11	14	17	19
1	***0***	***0***	24	25	24	23	24
0	***1***	***0***	1	1	1	1	1
0	***0***	***1***	0	0	0	0	0
1	***1***	***0***	6	9	9	10	10
1	***0***	***1***	3	2	2	1	3
0	***1***	***1***	0	0	0	0	0
1	***1***	***1***	16	13	11	9	4

Suppl Figure 1. Diagram representing histological structures of a growing follicle in avian feathered skin, adapted from Lucas and Stettenheim (1972)[29]. The left diagram shows a longitudinal section. The right diagrams represent a closer view of the differenciating feather epidermis at three levels, A, B and C.

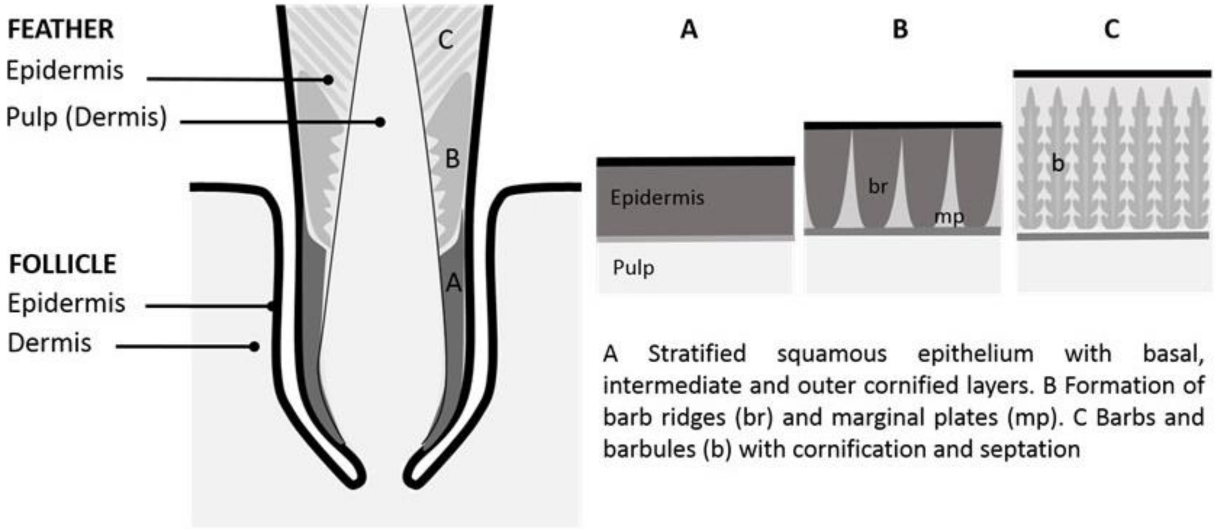

Suppl. Figure 2. Comparison of Viral RNA load between two extraction methods with and without proteinase K on feathers from experimentally H5N8-infected ducks (infected group, n=5 for each time point).

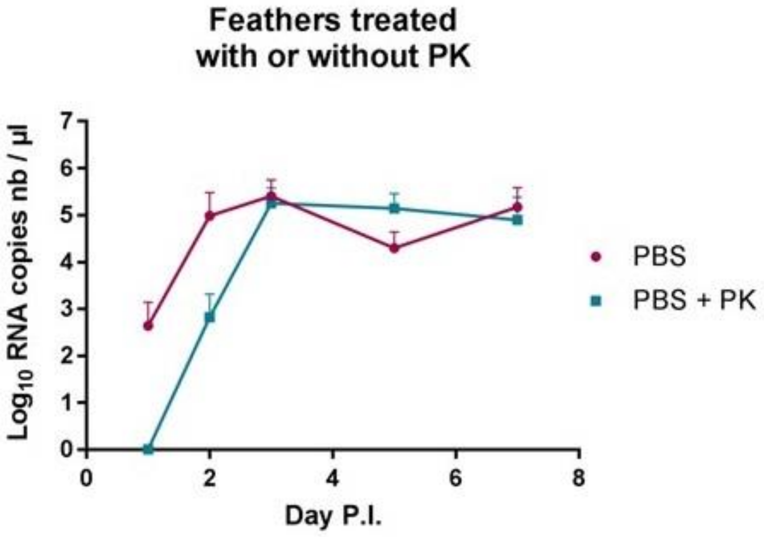

Suppl Figure 3. Comparison of droplet-digital *versus* real-time quantitative reverse transcription PCR for detection of AIV M gene on Flock #2. Comparison at the individual (Panel A) or flock level (Panel B). Results are expressed as $\log_{10}$ viral RNA copies/µL.

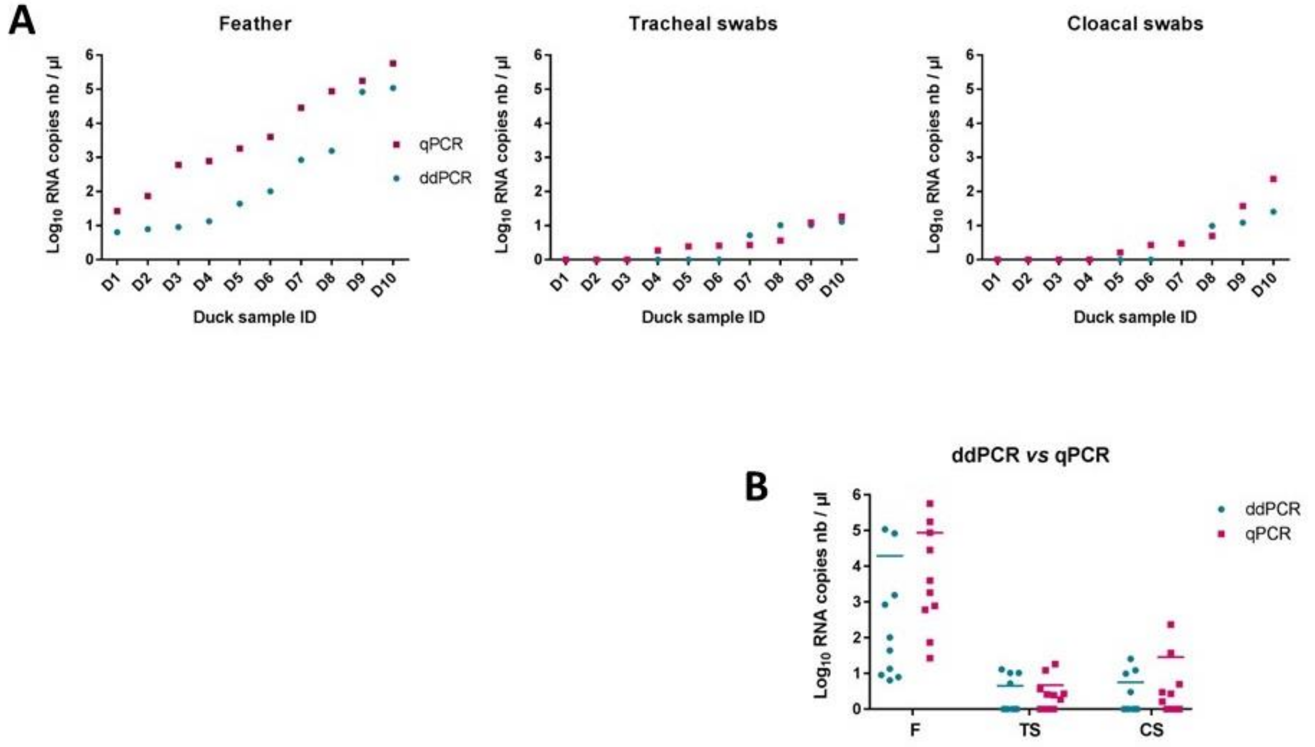

Supplement: Supplementary file 1 — Supplementary Information. [file 41598_2021_85109_MOESM1_ESM.pdf]
